# Toward Personalized Response Monitoring in Melanoma Patients Treated with Immunotherapy and Target Therapy

**DOI:** 10.3390/diagnostics15233054

**Published:** 2025-11-29

**Authors:** Federico Venturi, Elisabetta Magnaterra, Alberto Gualandi, Biagio Scotti, Carlotta Baraldi, Aurora Maria Alessandrini, Leonardo Veneziano, Elena Maria Cama, Barbara Melotti, Paola Valeria Marchese, Daniela Tassone, Simone Ribero, Marco Ardigò, Emi Dika

**Affiliations:** 1 Department of Medical and Surgical Sciences (DIMEC), Alma Mater Studiorum, University of Bologna, 40138 Bologna, Italy; 2 Oncologic Dermatology Unit, IRCCS Azienda Ospedaliero-Universitaria di Bologna, 40138 Bologna, Italy; 3 Oncology Unit, IRCCS Azienda Ospedaliero-Universitaria di Bologna, 40138 Bologna, Italy; 4 Plastic Surgery, IRCCS Azienda Ospedaliero-Universitaria di Bologna, 40138 Bologna, Italy; 5 Department of Medical Science, Dermatology Clinic, University of Turin, 10126 Turin, Italy; simone.ribero@unito.it; 6 Dermatology Unit, IRCCS Humanitas Research Hospital, Rozzano, 20089 Milano, Italy; 7 Department of Biomedical Sciences, Humanitas University, Pieve Emanuele, 20072 Milano, Italy

**Keywords:** melanoma, immunotherapy, target therapy, treatment related adverse events, ctDNA, microRNA, liquid biopsy, interferon signature

## Abstract

**Background/Objectives:** Immunotherapy and targeted therapy have revolutionized the treatment of advanced cutaneous melanoma. However, predicting individual response and managing resistance remain major challenges. This narrative review aims to evaluate the prognostic and predictive value of treatment-related adverse events (TRAEs) and circulating biomarkers—including lactate dehydrogenase (LDH), circulating tumor DNA (ctDNA), and microRNAs (miRNAs)—in anticipating therapeutic outcomes and personalizing treatment strategies. **Methods:** A comprehensive literature search was conducted across PubMed, Scopus, and Web of Science for studies published between January 2010 and September 2025. Eligible studies included clinical trials, observational cohorts, and translational research evaluating biomarkers or toxicity profiles in melanoma patients receiving immune checkpoint inhibitors or BRAF/MEK inhibitors. Emphasis was placed on dynamic indicators of treatment efficacy and integrative modeling approaches. **Results:** Evidence indicates that the emergence of low-to-moderate grade TRAEs—especially immune-related events like vitiligo, thyroiditis, and rash—is positively associated with response to immunotherapy. Similarly, pyrexia and dermatologic toxicities may correlate with outcomes under BRAF/MEK inhibition. ctDNA clearance within 6–12 weeks of therapy strongly predicts durable response and precedes radiologic changes. Specific miRNAs (e.g., miR-21-5p, miR-146a-5p) demonstrate dynamic modulation during treatment and may signal response or resistance. Interferon-driven gene expression profiles further stratify tumors into “hot” or “cold” immune phenotypes, refining predictive accuracy. **Conclusions:** Integrative models combining TRAEs, ctDNA, miRNA signatures, and interferon-related gene expression offer a multi-dimensional framework for early, individualized response monitoring. Prospective validation, harmonization of assays, and incorporation into adaptive clinical workflows are key to translating these insights into personalized melanoma care.

## 1. Introduction

Cutaneous melanoma remains one of the most aggressive forms of skin cancer, accounting for the majority of skin cancer-related deaths. In the United States, cutaneous melanoma comprises over 90% of melanoma cases and is associated with thousands of deaths annually, with mortality rates far exceeding those of nonmelanoma skin cancers, which rarely result in death [1,2,3,4,5,6,7,8,9,10]. In recent years, the therapeutic landscape has been transformed by the advent of immune checkpoint inhibitors (ICIs) targeting PD-1/PD-L1 and CTLA-4, as well as targeted therapies against BRAF and MEK in patients harboring BRAF mutations [11,12,13,14]. Despite these advances, not all patients respond equally, and durable benefit is limited to a subset. As a result, there is a pressing need for reliable predictive biomarkers to guide treatment decisions and personalize care [15]. Traditional predictors such as baseline tumor burden, lactate dehydrogenase (LDH) levels, and performance status offer limited granularity. Recently, attention has turned to on-treatment indicators, including the emergence of treatment-related adverse events (TRAEs), which may reflect immune system activation or off-target effects associated with efficacy. In parallel, circulating biomarkers such as ctDNA and microRNAs (miRNAs) are being explored as minimally invasive tools to monitor treatment response in real time. This review evaluates the potential of both toxicity profiles and circulating biomarkers to serve as early predictors of response and long-term outcomes in melanoma patients.

## 2. Materials and Methods

**Figure 1:**
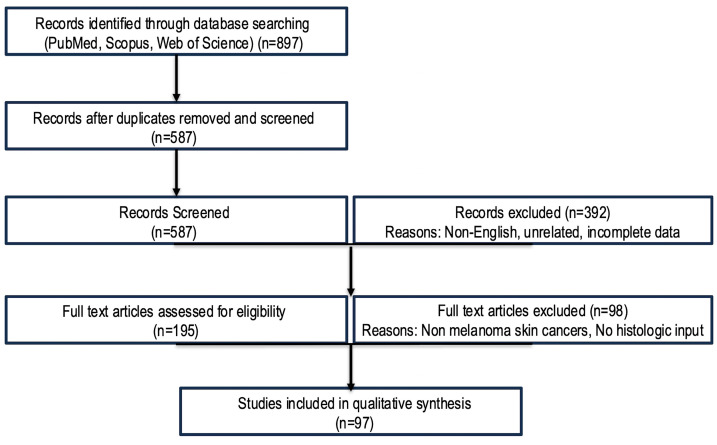
Flow diagram summarizing the identification, screening, eligibility, and inclusion process for studies analyzed. A comprehensive search was conducted across PubMed, Scopus, and Web of Science from January 2010 to September 2025. A total of 897 records were initially identified, of which 587 remained after duplicate removal. Following title and abstract screening, 195 full-text articles were evaluated for eligibility. Studies were excluded if they were non-relevant, duplicates, preclinical, or lacked data on treatment-related adverse events, circulating biomarkers, or immune transcriptomic signatures. Finally, 97 studies meeting the inclusion criteria were included in the qualitative synthesis.

This narrative review was conducted to comprehensively synthesize current evidence on clinical, molecular, and immunologic biomarkers for treatment response prediction and monitoring in patients with cutaneous melanoma treated with ICIs and targeted therapies. A comprehensive literature search was performed across PubMed/MEDLINE, Scopus, and Web of Science databases for studies published between January 2010 and September 2025. The search strategy combined both Medical Subject Headings (MeSH) and free-text terms related to the main concepts of the review: “melanoma,” “immune checkpoint inhibitors,” “targeted therapy,” “immune-related adverse events,” “BRAF/MEK inhibitors,” “circulating tumor DNA,” “microRNA,” “liquid biopsy,” “interferon signature,” “immune-inflamed tumor,” “predictive biomarkers,” and “treatment response.” Eligible studies included original research articles (prospective or retrospective), randomized controlled trials, meta-analyses, and significant mechanistic or translational studies investigating biomarkers of treatment response, resistance, or toxicity in melanoma. Exclusion criteria included case reports, editorials, non-peer-reviewed preprints, and animal-only studies unless they provided mechanistic insights directly supporting clinical findings. Only studies in English were considered. Reference lists of key reviews and seminal articles were manually screened to identify additional relevant publications. Studies were critically appraised for sample size, methodology, reproducibility, and translational impact. Preference was given to large multicenter studies, prospective biomarker validation cohorts, and high-impact mechanistic investigations (Figure 1).

## 3. Results

### 3.1. Treatment-Related Adverse Events (TRAEs) and Their Association with Therapeutic Efficacy

The development of TRAEs has emerged as a potential surrogate marker of therapeutic efficacy in melanoma, reflecting on-target biological activity and systemic immune engagement. Among these, immune-related adverse events (irAEs) associated with immune checkpoint inhibitors (ICIs) and targeted therapy–related adverse events (ttAEs) from MAPK pathway inhibition represent two mechanistically distinct but clinically informative phenomena. IrAEs arise from nonspecific activation of the immune system due to PD-1/PD-L1 or CTLA-4 blockade. Numerous retrospective and prospective studies have demonstrated a positive correlation between the occurrence of irAEs and improved clinical outcomes, including higher objective response rates, longer progression-free survival (PFS), and overall survival (OS) [16,17,18,19,20]. In a retrospective cohort of 49 melanoma patients treated with anti-PD-1 monotherapy, Wu et al. found that patients experiencing grade 1–2 irAEs achieved longer median progression-free survival (4.6 vs. 2.5 months; HR 0.52) and a trend toward improved overall survival (15.2 vs. 5.7 months; HR 0.50) compared with those without irAEs [18]. A larger meta-analysis in metastatic melanoma (*n* = 1474) reported that patients with any irAEs had superior overall survival (median 15.24 vs. lower) compared to those without; the authors estimated a substantial survival advantage in the irAE+ subgroup [20]. More recently, a pan-tumor prospective study further noted a positive correlation between irAEs and improved survival metrics, pointing to the reproducibility of the association beyond melanoma alone [21]. Cutaneous manifestations—such as vitiligo, rash, or pruritus—are among the most frequent and are particularly relevant in melanoma due to antigenic overlap between melanoma cells and normal melanocytes [22,23,24,25,26,27]. The appearance of vitiligo during anti-PD-1 or combination immunotherapy has been consistently associated with robust and durable responses, suggesting a shared immunological mechanism of melanocyte destruction [28,29,30]. Similarly, endocrine irAEs such as thyroiditis or hypophysitis, and gastrointestinal or hepatic toxicities, have been linked to favorable outcomes when adequately managed [31,32,33,34]. Importantly, the timing and grade of irAEs also appear relevant: early-onset, low-to-moderate toxicities may signal effective immune activation, whereas severe or multi-organ irAEs can necessitate treatment discontinuation, potentially offsetting clinical benefit [35,36]. Despite this association, the predictive value of irAEs is not absolute, and their absence does not preclude response. The interplay between immune activation, host genetics (e.g., HLA polymorphisms, cytokine gene variants), and gut microbiota composition may further modulate both efficacy and toxicity [37,38,39].

In parallel, ttAEs observed with BRAF and MEK inhibitors—including pyrexia, rash, arthralgia, photosensitivity, and laboratory abnormalities such as elevated liver enzymes—have also shown potential predictive implications. Pyrexia is among the most common toxicities in patients treated with dabrafenib plus trametinib, affecting up to 59% of patients (all grades) [40,41]. Some analyses suggest that the occurrence of pyrexia may correlate with better outcomes [42,43,44,45,46]. Atkinson et al. reported improved responses in patients experiencing pyrexia under BRAFi/MEKi regimens, suggesting pyrexia might act as a pharmacodynamic biomarker [47]. Earlier ASCO results posited that pyrexia was associated with more durable responses under combination BRAF/MEK therapy. Similarly, Schaefer et al. devised a pyrexia score algorithm combining C-reactive protein (CRP), LDH, leukocyte, and platelet counts; they showed that this score differentiated patients who developed pyrexia, and that systemic biomarker shifts (e.g., increase in CRP, fall in leukocytes/platelets) correlated with the inflammatory response induced by therapy [48,49]. However, the relationship is not uniform: in a study of patients with metastatic BRAF V600E/K melanoma, Menzies et al. observed that while pyrexia was frequent and recurrent under dabrafenib + trametinib, it was not statistically associated with baseline characteristics or drug efficacy, cautioning against overinterpretation [40].

Beyond pyrexia, rash and photosensitivity—common dermatologic toxicities—have been analogized in other kinase inhibitor settings (e.g., EGFR inhibitors) as correlates of adequate target inhibition, though direct evidence in melanoma/triose MAPK contexts remains limited [50,51]. Maculopapular rashes typically appear within the first few weeks of therapy and may be pruritic or desquamative. In a pooled analysis, their development was associated with better disease control and, in some cohorts, longer PFS [52,53]. Photosensitivity reactions, more commonly linked to vemurafenib, can significantly impact patient quality of life and adherence. These cutaneous reactions may represent a marker of on-target inhibition in skin tissues, paralleling drug activity in the tumor microenvironment. Optimal supportive care, including sun protection and topical corticosteroids, is essential to maintain treatment adherence [54]. Unlike irAEs, these toxicities arise from on-target inhibition of the MAPK pathway in normal tissues. Nevertheless, ttAEs can also compromise adherence and quality of life, highlighting the importance of proactive management and dose optimization to sustain long-term benefit [55,56].

The biological distinction between immune-driven and kinase-driven toxicities underscores the heterogeneity of TRAE mechanisms—yet both may act as dynamic indicators of drug–target interaction and patient immune or metabolic responsiveness. Collectively, evidence suggests that the emergence, timing, and severity of TRAEs—whether immune- or targeted-therapy-related—provide valuable, noninvasive clues to therapeutic efficacy. Integrating these clinical observations with molecular and serologic biomarkers (e.g., ctDNA kinetics, cytokine signatures, and microRNA profiles) could enhance early stratification of responders versus non-responders. Prospective, standardized analyses are required to validate TRAEs as functional biomarkers and to define how they can be integrated into predictive algorithms guiding individualized melanoma therapy.

### 3.2. Circulating Biomarkers

Circulating biomarkers—including conventional serologic markers (LDH, S100B), circulating tumor DNA (ctDNA), and small non-coding RNAs such as microRNAs (miRNAs)—offer complementary information that can anticipate therapeutic efficacy or resistance long before radiologic evidence becomes apparent. These biomarkers reflect different layers of tumor and host interaction: metabolic burden, genomic evolution, and immune modulation, respectively [57].

#### 3.2.1. Serum LDH and S100B

Serum lactate dehydrogenase (LDH) remains one of the most established and clinically implemented biomarkers in melanoma [58,59]. Elevated baseline LDH is incorporated into the AJCC 8th edition staging system as an indicator of poor prognosis, correlating with increased tumor burden, hypoxia-driven glycolytic reprogramming, and aggressive disease biology [9,60]. Multiple studies and meta-analyses have confirmed that elevated LDH predicts shorter PFS and OS in patients treated with both ICIs and BRAF/MEK inhibitors [61,62]. However, the utility of LDH as a dynamic biomarker remains limited due to its lack of specificity and delayed kinetics. Fluctuations may reflect necrosis or inflammation rather than true disease progression [63]. Similarly, S100B, a calcium-binding protein expressed by melanocytes and glial cells, has been validated as a sensitive marker for disease activity and relapse [64,65,66]. Rising S100B during therapy correlates with disease progression, whereas sustained decreases are associated with response. Nonetheless, S100B’s sensitivity for early response detection remains modest, particularly in patients with low tumor burden or isolated organ metastases (e.g., CNS or subcutaneous sites) [67,68,69].

#### 3.2.2. Circulating Tumor DNA (ctDNA)

The emergence of ctDNA as a real-time, quantitative measure of tumor burden has profoundly changed melanoma monitoring [70]. ctDNA, comprising short DNA fragments released from apoptotic or necrotic tumor cells, enables both quantification of tumor load and molecular profiling of resistance mechanisms [71,72]. The detection and dynamics of ctDNA during therapy have shown remarkable correlation with clinical outcomes. Several studies have demonstrated that early ctDNA clearance predicts durable response to immune checkpoint inhibition [73,74,75]. Lee et al. (Nat Med 2020) reported that patients achieving undetectable ctDNA within 12 weeks of anti-PD-1 therapy had significantly longer OS and PFS compared to those with persistent ctDNA [76]. Similarly, Seremet et al. found that a ≥90% decrease in ctDNA at week 6 predicted radiologic response with high specificity, outperforming LDH and radiographic criteria [77]. Under targeted therapy, the dynamics are even more pronounced [75,78,79]. Conversely, ctDNA reappearance often heralds progression weeks before clinical relapse, highlighting its potential as an early resistance marker [80]. Technological advances, such as ultrasensitive digital PCR and next-generation sequencing (NGS) platforms (e.g., CAPP-Seq, BEAMing, and ddPCR), have enabled detection of ctDNA at variant allele frequencies below 0.1% [81]. This sensitivity allows not only monitoring of treatment response but also identification of emerging resistance mutations, such as NRAS or MEK1/2 alterations during BRAF/MEK inhibition, or PTEN loss and β2-microglobulin mutations under ICI therapy [82]. Furthermore, recent multi-omic approaches integrating ctDNA with circulating tumor cells (CTCs) and exosomal RNA signatures provide complementary insights into tumor heterogeneity and immune escape dynamics. Despite these advances, standardization of ctDNA assays remains a major challenge. Differences in pre-analytical handling, sequencing depth, and threshold definitions limit inter-study comparability. Harmonization efforts—such as those proposed by the European Liquid Biopsy Society (ELBS)—are ongoing to establish reference standards and facilitate ctDNA incorporation into clinical trials and real-world response monitoring. These initiatives aim to define pre-analytical and analytical workflows (e.g., blood collection, processing, sequencing), improve inter-laboratory comparability through ring trials, and build consensus on clinical validity and utility endpoints to support ctDNA use as a surrogate biomarker of treatment response [83,84]. The Association for Molecular Pathology and the College of American Pathologists have issued joint consensus recommendations emphasizing the need to report key pre-analytical variables, assay performance metrics, and validation procedures to ensure clinical utility and reproducibility of ctDNA assays [85].

#### 3.2.3. Circulating MicroRNAs (miRNAs)

Among novel circulating biomarkers, miRNAs have gained increasing attention for their potential to mirror both tumor-intrinsic biology and host immune dynamics [10]. These small, non-coding RNAs regulate post-transcriptional gene expression and are remarkably stable in serum, plasma, and extracellular vesicles. In melanoma, deregulated miRNA profiles are associated with tumor progression, metastatic potential, and immune modulation [86,87,88,89,90,91]. Several circulating miRNAs have been implicated as predictors of response to ICIs and targeted therapies [92,93]. For example, elevated baseline levels of miR-21-5p and miR-146a-5p—two key regulators of NF-κB-dependent inflammatory signaling—have been associated with adverse prognosis and resistance to immunotherapy [87]. Dynamic downregulation of these miRNAs during treatment correlates with favorable response, suggesting they reflect tumor-immune equilibrium. Conversely, upregulation of miR-155-5p and miR-34a has been linked to enhanced T-cell activation and longer PFS under anti-PD-1 therapy [94]. Other studies have shown that exosomal miRNAs such as miR-125b or miR-31-5p may predict early progression, while miR-16 and miR-223 correlate with immune-related toxicity, hinting at shared regulatory networks between efficacy and irAEs [95,96]. The integration of exosomal miRNA profiling offers a promising step forward: exosomes protect miRNAs from degradation and originate from both tumor and immune cells, making them reflective of tumor–host crosstalk. In a 2023 study, De Martino et al. identified a three-miRNA plasma signature (namely hsa-miR-200c-3p, hsa-miR-144-3p and hsa-miR-221-3p) that were differentially expressed in plasma-derived exosomes from melanoma patients and controls [97]. Despite these promising data, translational application is still hampered by technical variability (sample processing, normalization, platform heterogeneity) and lack of universally accepted reference miRNAs. Harmonized pipelines and validation in large, prospective cohorts are essential to move these signatures from discovery to clinical use. An emerging frontier involves the integration of multiple circulating biomarkers into composite predictive frameworks. Combined analysis of ctDNA kinetics, LDH levels, and specific miRNA expression patterns improves sensitivity and specificity for early response detection compared to any single biomarker. Similarly, machine-learning algorithms incorporating ctDNA, cytokine panels, and immune cell profiling have been proposed to generate personalized “response probability scores,” opening the way for adaptive treatment strategies.

### 3.3. Hot and Cold Tumors: Interferon Signatures and Immune-Inflamed Phenotypes

Melanomas (and more broadly solid tumors) can be broadly stratified into “hot” (immune-inflamed/T cell-infiltrated) and “cold” (immune-excluded or immune-desert) phenotypes, which differ fundamentally in their immune microenvironment, transcriptional programs, and responsiveness to immune checkpoint blockade [98]. Key to this stratification is the presence or absence of interferon (IFN)-driven gene expression signatures that reflect active immune engagement within the tumor microenvironment (TME). A seminal work by Ayers et al. introduced a T cell-inflamed gene expression profile (GEP) centered on IFN-γ-responsive genes (e.g., CXCL9, CXCL10, IDO1, STAT1, GZMB) that correlated with response to anti-PD-1 therapy across multiple tumor types, including melanoma [99]. Their analysis showed that baseline expression of this IFN-γ signature predicted clinical benefit better than PD-L1 immunohistochemistry alone, and the signature was subsequently refined and validated in larger cohorts. Subsequent studies in melanoma and pan-cancer cohorts have reinforced the centrality of IFN signaling in distinguishing “hot” from “cold” tumors. Grasso et al. (2020) demonstrated that conserved IFN-γ signaling and T-cell infiltration were among the strongest correlates of clinical response to checkpoint inhibitors, further validating the relevance of IFN signatures [100]. The multi-omic profiling of checkpoint-treated melanoma by Newell et al. confirmed that “hot” tumors are characterized by elevated expression of IFN-γ-responsive genes, abundant CD8+ T cells, and PD-L1 upregulation, while “cold” tumors show suppression of these immune modules [101]. More recently, the Dual Role of Interferon-Gamma in Melanoma was reviewed by Wawrzyniak & Hartman, who emphasize that IFN-γ is not a purely beneficial mediator: although its presence is often favorable (driving antigen presentation, T-cell recruitment, and cytotoxicity), chronic or dysregulated IFN signaling may contribute to immune exhaustion or upregulation of resistance pathways (for instance JAK/STAT pathway defects) that blunt responsiveness to therapy [102]. Likewise, Brockwell et al. reviewed how type I interferons (IFN-α/β) act upstream to prime the innate immune milieu and support the transition to a T cell-inflamed phenotype, thereby promoting responses to checkpoint inhibitors [103]. Beyond static baseline classification, dynamic changes in IFN signature—and shifts from “cold” to “hot” over time—have prognostic significance [102,104]. For instance, the DONIMI trial used a baseline IFN-γ signature to stratify melanoma patients for neoadjuvant immunotherapy, demonstrating that patients with high IFN-γ scores derived benefit from PD-1 monotherapy, whereas low-score tumors might require intensified therapy [105]. In that trial, tumors that converted from IFN-γ-low to -high early under therapy showed a higher rate of pathologic response, indicating that temporal induction of IFN signaling may act as a biomarker of treatment engagement. Another layer of nuance comes from spatially informed gene signatures. A recent study partitioned gene expression by the spatial compartments defined by CD68+ macrophages, CD45+ leukocytes, and tumor cells and found that the strongest predictive signals of response came from genes enriched in IFN and immune pathways within these immune niches, underscoring the importance of local IFN–immune interactions rather than bulk tumor averages [106]. Furthermore, a recent study proposed an ITRGM signature to stratify melanoma patients into immune-hot vs. immune-cold categories, showing that high signature scores correlated with increased immune cell infiltration, tumor mutational burden, and better immunotherapy response across multiple cohorts [107]. Mechanistically, Holzgruber et al. showed that type I interferon signaling in melanoma cells can induce PD-1 expression via the IFNAR–JAK/STAT axis, which has implications for how tumor-intrinsic IFN pathways modulate checkpoint receptor expression and response to therapy [108]. This highlights that both tumor-cell intrinsic and immune-compartment IFN signaling intersect to define the immune phenotype of melanoma, with potential feedback loops influencing ICI sensitivity or resistance.

**Figure 2:**
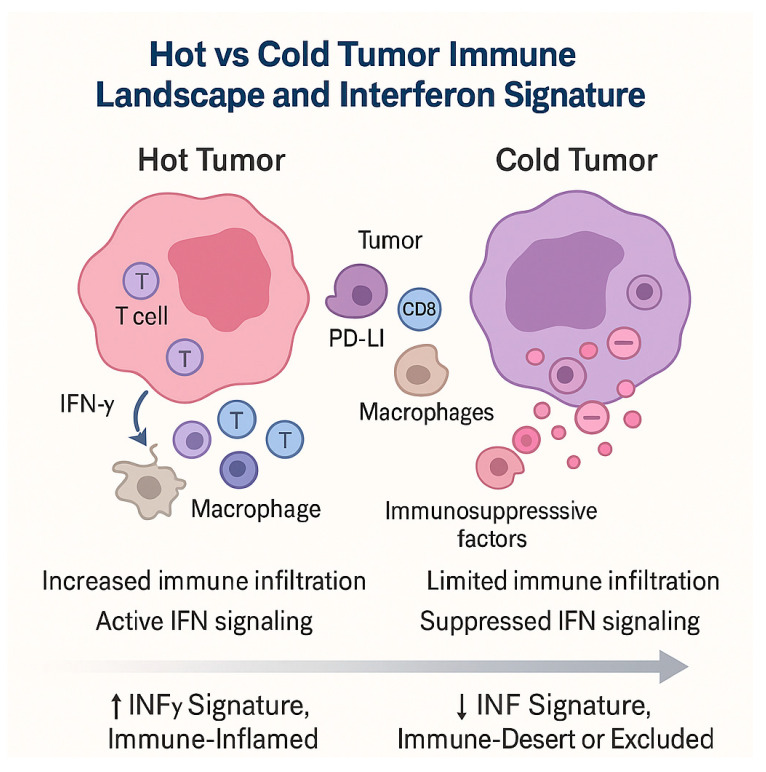
Representation of the immunologic spectrum in melanoma.

Collectively, the literature supports a refined view of the hot vs. cold tumor paradigm (Figure 2):

- Hot tumors: enriched in IFN-γ-driven transcripts, T cell infiltration, chemokine expression, antigen presentation machinery, and adaptive immune activation—generally more responsive to ICIs.
- Cold tumors: deficient in IFN signature, with limited immune infiltrate, exclusion of effector T cells, and immunosuppressive barriers—often refractory to monotherapy.
- Dynamic conversion: Some tumors initially cold may “heat up” under therapy, with induced IFN signaling and immune infiltration signaling favorable biological response.
- Dualistic roles: IFN signatures can also be modulated by resistance mechanisms (e.g., JAK mutations, chronic IFN exposure, induction of inhibitory pathways), complicating their predictive utility.

Incorporating baseline IFN signatures, spatially mapped immune-IFN modules, and dynamic IFN induction into predictive models may therefore deepen our ability to stratify patients, tailor immunotherapy combinations, and monitor early immune activation in melanoma.

## 4. Discussion

The assessment of treatment response in melanoma is increasingly driven by integrative models that combine clinical, molecular, and immune-derived data, moving beyond single-parameter predictors. Recent studies demonstrate that multidimensional approaches—incorporating imaging techniques, toxicity profiles, circulating biomarkers (such as cell-free DNA, RNA, proteins, and immune cell subsets), and tumor immune signatures—enable more accurate, real-time, and personalized prediction of response to ICIs [109,110,111,112,113,114,115,116]. Clinical models that utilize routine parameters (e.g., performance status, metastatic sites, serum lactate dehydrogenase, neutrophil–lymphocyte ratio) have shown moderate predictive value for response and survival in metastatic melanoma treated with anti-PD-1 ± ipilimumab, with validated nomograms available for clinical decision support [117,118]. Molecular profiling, including genomic, transcriptomic, and immune signatures from tumor tissue, further refines prediction, identifying features such as tumor mutational burden, antigen presentation machinery, and immune cell infiltration as key determinants of response [110,111,119,120,121,122]. Immune-derived data, particularly the characterization of the tumor microenvironment (TME) and peripheral immune cell populations (e.g., circulating PD-1+ CD4+ effector memory T cells), have emerged as independent predictors of progression-free survival and response to ICIs [112,113]. Integrative models that combine these data types outperform single biomarkers and facilitate patient stratification, therapy selection, and monitoring for resistance or toxicity [121,123,124]. This comprehensive framework supports precision medicine in melanoma, allowing clinicians to tailor therapies and optimize outcomes by leveraging multidimensional, real-time data (Table 1).

The findings summarized in this review support a growing paradigm shift in melanoma care—from static, baseline predictors to dynamic, integrative monitoring strategies. Across the included studies, several key themes emerged.

First, the consistent association between low-to-moderate grade TRAEs (particularly vitiligo, thyroiditis, and rash) and treatment response across retrospective and prospective cohorts reinforces their role as on-treatment indicators of immune engagement [125,126,127,128,129,130]. However, most studies were observational and variably reported toxicity grading, warranting caution in interpretation and highlighting the need for standardized toxicity-response models.

Second, ctDNA dynamics—particularly early clearance—demonstrated strong and re-producible correlation with progression-free and overall survival. Several studies used orthogonal validation with radiologic and clinical endpoints, enhancing the robustness of ctDNA as a biomarker. Nevertheless, technical heterogeneity in ctDNA platforms (e.g., ddPCR vs. NGS) remains a major barrier to widespread clinical adoption [78,131,132,133,134,135].

Third, circulating miRNA signatures offered nuanced insights into tumor-immune crosstalk, though the current evidence base is limited by small sample sizes and variability in profiling techniques. Validation in larger, prospective cohorts is essential [136,137,138].

Lastly, interferon-related gene expression profiling (IFN-GEP) emerged as a powerful predictor of ICI response, stratifying tumors into hot vs. cold phenotypes. Its clinical relevance has been demonstrated in both retrospective analyses and prospective stratification trials (e.g., DONIMI). However, the dual role of IFN signaling in both activation and resistance underscores the need for context-aware interpretation [100,102,139,140,141].

Taken together, these findings support a framework in which toxicity patterns, liquid biopsy metrics (ctDNA, miRNAs), and transcriptomic signatures (IFN-GEP) are integrated into early, individualized response monitoring algorithms. Future research should focus on harmonizing assay methodologies, validating composite biomarkers across treatment modalities, and translating these insights into adaptive clinical workflows that can truly personalize melanoma care.

Despite the promising findings reported across studies, several limitations must be acknowledged. First, the underlying evidence is largely based on retrospective analyses, early-phase prospective cohorts, or biomarker sub-studies with limited sample sizes and heterogeneous patient populations. This heterogeneity, along with variability in treatment regimens, follow-up intervals, and outcome definitions, may limit the generalizability of results. Additionally, potential publication bias cannot be excluded, as negative studies are less frequently reported. Second, our review is narrative in nature: while supported by a structured search strategy, we did not perform a formal risk-of-bias assessment nor a meta-analytic synthesis of effect sizes. These limitations should be considered when interpreting the clinical relevance of candidate biomarkers.

Future research should prioritize the prospective validation of promising on-treatment biomarkers such as low-grade immune-related adverse events (e.g., vitiligo, thyroiditis), ctDNA dynamics, and miRNA expression profiles. Integration of these biomarkers into stratified clinical trial designs may help define composite predictors of response. Large-scale, multi-center studies with harmonized methodologies and real-time sampling will be essential to confirm their predictive utility. Additionally, longitudinal multi-omics profiling may offer insights into the temporal evolution of treatment response and resistance mechanisms.

The convergence of clinical, molecular, and immune-derived data is redefining how treatment response in melanoma is assessed. Rather than relying on single-parameter predictors, integrative models that combine toxicity profiles, circulating biomarkers, and tumor immune signatures provide a multidimensional framework for real-time and personalized response prediction.

## 5. Conclusions

**Figure 3:**
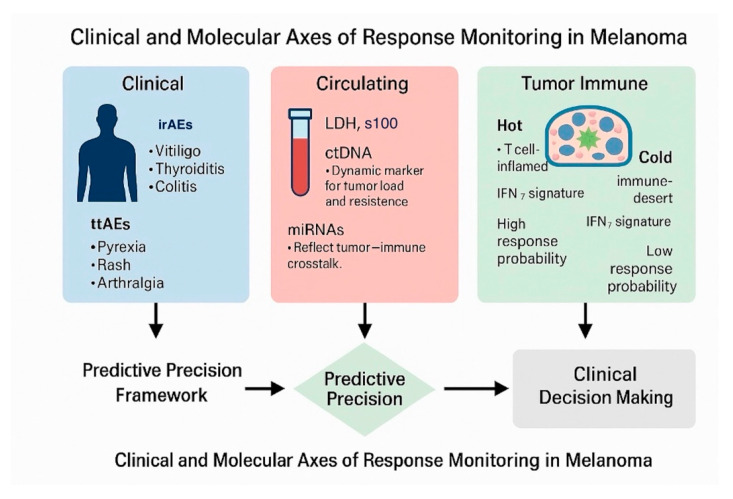
Algorithm for Personalized Response Monitoring in Melanoma. This dynamic, multi-parametric algorithm integrates clinical toxicity patterns, liquid-biopsy biomarkers, and tumor immune signatures into a unified precision-response monitoring framework. It supports early, individualized therapeutic adaptation aimed at maximizing efficacy while minimizing toxicity.

The integration of clinical, molecular, and immune-derived biomarkers is reshaping how therapeutic efficacy is monitored and predicted in melanoma. TRAEs, particularly immune-related toxicities, provide valuable real-time insight into immune activation; while circulating biomarkers such as ctDNA and miRNAs offer dynamic and minimally invasive indicators of tumor burden and biological response. In parallel, interferon-driven gene expression profiles and immune-inflamed (“hot”) tumor signatures have emerged as robust determinants of checkpoint inhibitor sensitivity, bridging the gap between tumor-intrinsic features and host immune competence. Collectively, these complementary dimensions—clinical, serological, and transcriptomic—form the foundation for integrative predictive models capable of anticipating treatment outcomes with unprecedented precision (Figure 3). Although not the primary focus of this review, it is important to acknowledge that standard imaging modalities such as PET/CT and MRI remain integral components of treatment monitoring algorithms. These imaging tools are already embedded in current follow-up protocols and are routinely employed to assess disease burden, detect progression, or confirm radiological responses. For this reason, they were not explicitly discussed in detail here, as our goal was to emphasize emerging, complementary predictors that may enhance or personalize treatment monitoring. Nonetheless, the proposed approach is meant to be integrative, and imaging findings should be interpreted alongside toxicity profiles and molecular biomarkers, in line with established guidelines.

While the algorithm presented here is primarily tailored to melanoma, its conceptual framework—based on dynamic, multimodal predictors such as immune-related toxicities, liquid biopsy dynamics, and transcriptomic signatures—may be broadly applicable across other malignancies [130,132]. For example, immune-related thyroid dysfunction has been correlated with treatment response in non-small cell lung cancer (NSCLC), while early clearance of ctDNA has shown predictive value in colorectal and breast cancers. Several tumor types are now being investigated in prospective trials using composite biomarkers to guide immunotherapy or targeted therapy response. These analogies underscore the potential for a cross-disciplinary approach to personalized response monitoring, and suggest that similar biomarker- and toxicity-based models could be adapted and validated beyond the melanoma setting.

The convergence of these multi-omic indicators within artificial intelligence-based frameworks will likely enable early identification of responders, optimization of therapeutic intensity, and avoidance of unnecessary toxicity. Ultimately, advancing toward personalized response monitoring in melanoma will depend on large-scale prospective validation, standardization of biomarker assays, and incorporation of longitudinal data into adaptive clinical workflows. Such multidimensional and dynamic approaches promise to transform melanoma management from reactive to proactive care, aligning therapeutic decisions with each patient’s unique biological and immune landscape (Table 1).

## Figures and Tables

**Table 1:** Stepwise algorithm summarizing the proposed clinical workflow.

**Step**	**Process**	**Key Indicators/Tools**	**Interpretation/Clinical Action**
Baseline profiling	Comprehensive clinical and molecular characterization before therapy initiation.	LDH, S100B, baseline ctDNA, miRNA panel (miR-21-5p, miR-146a-5p), PD-L1 IHC, IFN-γ gene-expression profile (GEP), tumor mutational burden (TMB).	Stratify tumors as “hot” (IFN-high, inflamed) or “cold” (immune-desert). Define initial risk and expected treatment sensitivity.
Early on-treatment assessment (weeks 4–8)	Initial biological and clinical response evaluation.	Onset and grade of TRAEs (irAEs or ttAEs), repeat ctDNA quantification, miRNA modulation, early imaging if available.	Early ctDNA clearance ± low-grade irAEs → favorable immune activation; persistent ctDNA ± no toxicity → consider intensification or switch.
Dynamic monitoring (weeks 8–16)	Continuous assessment of therapeutic efficacy and resistance emergence.	Longitudinal ctDNA kinetics, serial miRNA profiles, cytokine panels, serial IFN-γ GEP measurements.	Declining ctDNA + induced IFN signature → sustained response; rising ctDNA + loss of IFN signature → emerging resistance.
Integrative modeling	Multivariate data integration to quantify individualized response probability.	Machine-learning model combining TRAEs, ctDNA, miRNA, LDH, and IFN-γ GEP.	Generates a Response Probability Score (RPS) categorized as high/intermediate/low.
Adaptive therapeutic decision	Adjust treatment intensity based on integrated signal.	Compare RPS trajectory with toxicity profile and radiologic data.	High RPS: continue current regimen. Low RPS + no irAEs: escalate (add CTLA-4 blockade or switch). Intermediate RPS: maintain with close follow-up.
Validation and feedback loop	Continuous model refinement and prospective data accrual.	Real-world registry data, digital pathology, radiomics integration.	Calibrate thresholds, improve predictive accuracy, and enable AI-assisted decision support for future patients.

## Data Availability

No new data were created or analyzed in this study.

## Abbreviations

The following abbreviations are used in this manuscript:

trAEs	treatment-related adverse events
irAEs	immune-related adverse events
ICIs	immune checkpoint inhibitors
ttAEs	Targeted therapy-related adverse events
LDH	lactate dehydrogenase
miRNAs	microRNAs
PFS	progression-free survival
OS	Overall survival
ctDNA	circulating tumor DNA
NGS	next-generation sequencing
ELBS	European Liquid Biopsy Society
GEP	gene expression profile
TME	tumor microenvironment
